# Aggio et al. Respond to “Lessons for Research on Cognitive Aging”

**DOI:** 10.1093/aje/kww030

**Published:** 2016-05-24

**Authors:** Daniel Aggio, Lee Smith, Abigail Fisher, Mark Hamer

We thank Dr. Belsky (1) for his interest in our study (2), in which we investigated associations between physical activity and cognitive function in young people. He raises several important issues on the role of physical activity as a public health strategy in the prevention of cognitive decline. The issue of “neuroselection,” wherein individuals with better cognitive function are more likely to engage in healthy behaviors such as physical activity and refrain from unhealthy ones such as smoking, is a potential source of bias in observational studies. The alternative hypothesis is that of “neuroprotection,” in which engagement in healthy behaviors is likely to lead to enhanced cognitive development.

We agree that confounding by pre-existing characteristics is a general problem in any epidemiologic work. Indeed, the children in our accelerometry sample typically came from wealthier families and had parents with higher levels of education than did those excluded from the analysis. Neuroselection may also explain the associations observed between physical activity and cognitive function. Longitudinal studies provide an opportunity to investigate whether cognitive function at baseline is associated with positive health behaviors in later life. For example, children with better cognitive function demonstrate higher fitness levels in adulthood (3). One potential mechanism may be that individuals with superior cognitive function are better able to interpret and respond to health advice than are individuals with lower cognitive function (4).

The basis of the neuroselection hypothesis is that individuals with higher cognitive function elect to live healthier lives. However, in our study (2), physical activity was measured at age 7 years, meaning that if the neuroselection argument were true, children with higher cognitive function would already be selecting to be active at this early age. Children's knowledge of healthy behaviors is limited in this age group (5), which suggests that young children may not be able to correctly identify healthy behaviors regardless of cognitive ability. Moreover, during early childhood, self-selection of healthy behaviors may be restricted because of the low levels of autonomy due to school policy and parental restrictions. Physical activity at this age is predominantly influenced by parents and the school environment (6, 7). Therefore, participation in healthy behaviors among young children is largely determined by their parents’ and teachers’ decisions rather than their own. Adolescents and young adults have high levels of independence and therefore the ability to respond to new information independently. Together, these points suggest that the process of neuroselection is more likely to occur from adolescence onwards than in childhood. Further longitudinal studies with multiple repeated measures of health behaviors and cognitive function throughout childhood and adolescence are needed to address this hypothesis.

Dr. Belsky raises an important point relating to the inconsistent effects of exercise on cognitive function that have been observed in recent randomized trials. This suggests that successful interventions may need to utilize physical activity in ways that also engage the brain. Indeed, this also emphasizes the main finding from our study, which was the importance of the context of physical activity and sedentary behavior in their associations with cognitive function. There is also research demonstrating the importance of context on other health outcomes. Notably, in a recent prospective study, childhood membership in a club was associated with a lower risk of mortality in adulthood that was independent of physical activity levels (8).

In summary, physical activity is a potential public health strategy for the prevention of neurodegenerative diseases, and early life may represent a crucial period for intervention. Further investigation on effects of specific types and domains of activity is warranted.
